# Synthetic cell preservation strategies enable their storage and activation at the point of use†

**DOI:** 10.1039/d5cc00826c

**Published:** 2025-04-29

**Authors:** Ignacio Gispert, Yuval Elani

**Affiliations:** a Department of Chemical Engineering, Imperial College London, South Kensington London SW7 2AZ UK; b Fabricell Imperial College London, South Kensington London SW7 2AZ UK y.elani@imperial.ac.uk

## Abstract

Synthetic cells encapsulating cell-free protein synthesis machinery are currently limited to laboratory use due to preservation challenges. They are typically produced and used immediately. We present drying methods for long-term ambient storage that preserve their integrity and functionality, enabling activation at the point-of-use, overcoming key barriers to their deployment in biotechnology.

## Abstract 2

We demonstrate strategies for preserving synthetic cells containing transcription-translation machinery *via* cold or dry storage. This enables point-of-use activation and broadens the applicability of synthetic cells beyond the laboratory.

## Graphical abstract

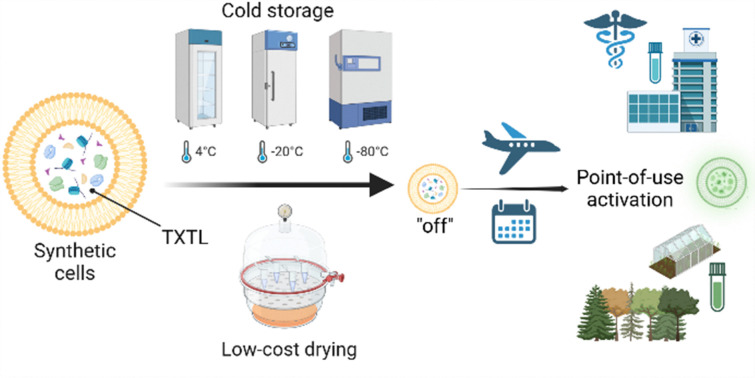

In recent years, synthetic cells have advanced from simple mimics of living cells – constructed by encapsulating biological components within lipid bilayer vesicles^1–3^ – to promising tools for drug delivery, biosensing, cellular therapeutic, diagnostic, and on-demand biomanufacturing applications.^4–6^ Such applications are made possible by incorporating transcription-translation (TXTL) systems containing the biochemical machinery of living cells (DNA, RNA polymerases, ribosomes, nucleotides, *etc*) in a cell-free environment. Nevertheless, despite the significant potential of synthetic cells,^7^ their current use is limited to highly specialised and controlled laboratory settings.

Synthetic cells are prone to functional decay and degradation due to oxidation, hydrolysis, and environmental factors such as temperature fluctuations.^8–10^ Exogenous chemicals and enzymes can also compromise the integrity of the lipid bilayer and the functionality of the encapsulated TXTL machinery.^11,12^

Finally, they have a limited operational lifetime; once activated, protein synthesis typically persists for only a few hours until essential building blocks are depleted, resulting in the cessation of protein production.

For applications beyond the laboratory, long-term synthetic cell storage in an inactive state would likely require refrigeration, freezing, or lyophilisation—methods that have not yet been established. An added layer of complication involves the logistical challenges associated with point-of-use applications in resource-limited settings, where cold-chain infrastructure is unavailable.

This situation is not new in the field of Synthetic Biology. Early work on cell-free TXTL faced similar challenges, which were remedied by the development of cell-free TXTL paper-based platforms,^13^ which enabled the development of paper-based circuits for point-of-use applications for the detection of water pollutants,^14–16^ viruses^17,18^ (including Zika^19^ or Covid-19^20^), or heavy metals^21,22^ with detection possible in complex matrices.^23,24^

Building on these advancements, we investigate the long-term ambient storage of synthetic cells, and not just cell-free solutions—an important step toward their real-world deployment. We present a simple and cost-effective strategy using low-cost drying techniques enabled by an inexpensive drying device, preserving the functionality of vesicle-based synthetic cells encapsulating DNA and cell-free TXTL machinery.

We hypothesised that long-term storage of synthetic cells could be achieved by freeze-drying (lyophilisation), as previously achieved with paper-based TXTL technologies.^13,25^ First, we considered how the freezing could affect the stability and functionality of the vesicles and their encapsulated cargo.

Cooling and freezing lipid bilayers reduce the membrane fluidity and increase their elastic modulus, with the lipid bilayers also undergoing dehydration. This affects the ability of membranes to withstand mechanical stresses, potentially compromising vesicle integrity during freezing.^26,27^ It is known, however, that the addition of sugars to the external media provides cryoprotection properties,^28,29^ preventing damage to the bilayer and leakage during freezing thanks to the formation of hydrogen bonds between the sugar and the lipid headgroups,^26^ a mechanism we leverage in our study.

Based on previous reports,^30^ we decided to make the synthetic cell membrane out of EggPC, a mixture of phosphatidylcholine lipids containing approximately 45% saturated lipids and ∼55% unsaturated lipids. We prepared osmotically-balanced EggPC synthetic cells encapsulating PURExpress TXTL (Fig. S1, ESI†) and their activity was assessed after storage in the fridge (4 °C), commercial freezer (−20 °C), or an ultra-low temperature freezer (−80 °C) with various cryoprotectants (Fig. 1A).

**Fig. 1:**
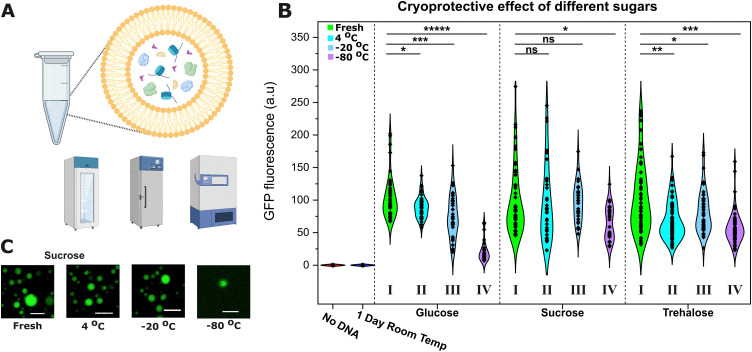
Synthetic cells can be preserved and activated after storage in cold conditions. (A) Schematic of a synthetic cell encapsulating TXTL cell-free protein synthesis machinery and the three devices employed in these experiments: a fridge, a commercial freezer and an ultra-low temperature freezer. (B) The cryoprotective effect of glucose, sucrose, and trehalose was determined as a measure of the protein expression after overnight storage in cold conditions. The violin plots show the distribution in fluorescence intensities obtained from microscopy images of over 40 synthetic cells in each condition. For each sugar, the GFP fluorescence of fresh samples (green, I) was normalised to 100 a.u. and the signals of samples stored in the fridge (4 °C, cyan, II), commercial freezer (−20 °C, blue, III), or ultra-low temperature freezer (−80 °C, purple, IV) were compared to the fresh sample to determine the level of activity after storage. With sucrose, no difference was found when stored at 4 °C or −20 °C. If TXTL was left for 1 day at room temperature and then activated, no GFP expression was seen due to functional degradation of TXTL components, demonstrating the need for preservation strategies. Statistical significance was calculated using an unpaired two-tailed t-test assuming unequal variance (*α* =  0.05). *P* value: ***** <0.00005, *** <0.0005, ** <0.005, * <0.05, ns = not significant. (C) Representative microscopy images of synthetic cells in sucrose after activation under different conservation scenarios. Size bar = 20 μm.

The next day, the samples were retrieved, imaged (Fig. 1C), and the constitutive expression of dasherGFP was recorded after 2 h of incubation at 37 °C and compared to the signals of fresh samples to measure the TXTL activity (Fig. 1B).

Sucrose was found to provide the best cryoprotection. Synthetic cells stored in sucrose (middle panel) in the fridge (4 °C, cyan, II) or in a commercial freezer (−20 °C, blue, III) expressed similar levels of GFP to fresh samples (control, green, I). However, with glucose (left) or trehalose (right), the synthetic cells did not maintain the same level of activity and saw significant 15–20% reductions in GFP fluorescence (unpaired *t*-test, *n* > 40). Storage in an ultra-low temperature freezer (−80 °C, purple, IV) caused a significant loss of activity with all the sugars, with sucrose achieving the best protection (35% reduction *vs.* 45 and 85% for trehalose and glucose respectively, unpaired *t*-test, *n* > 40).

We hypothesise that the reduction in expression is caused by a loss of some encapsulated cargo from the synthetic cell lumen to its environment during the freezing process. The optimal TXTL expression requires a precise stoichiometry (in this case, the 36 proteins of PURE plus the ribosomes, amino acids, rNTPs and tRNAs), hence small disturbances can have a large effect on the recorded GFP expression. Differences in osmotic pressure can cause the leakage of the encapsulated contents^31,32^ and a reduction in size (as observed in Fig. S2, ESI†), and during freezing, an osmotic gradient across the membrane forms.^33^ Despite the isosmotic sugar concentrations in our synthetic cells, trehalose and glucose provided worse protection than sucrose.

As anticipated, cooling was essential to preserve synthetic cell activity. Control experiments demonstrated that the TXTL machinery became inactive when left at room temperature overnight, showing a need for preservation strategies. The TXTL machinery was incubated in the absence of DNA at room temperature for 24 hours, and then, it was encapsulated inside synthetic cells together with DNA. Analysis of these synthetic cells showed that no GFP expression was detected (Fig. 1B, “1 Day Room Temp” sample). These results underscore the need of developing effective preservation for synthetic cells. We also demonstrated that the expression was not activated during the synthetic cell generation process itself (*i.e.*, prior to cooling and freezing), thus giving a false positive, as shown in Fig. S3 (ESI†); expression was only triggered following incubation at 37 °C.

These findings suggest synthetic cells could be employed at a point of use where appropriate resources are available. In these scenarios, synthetic cells could be prepared in a central facility and distributed under low-temperature conditions, leveraging existing cold supply chains and refrigeration techniques. However, not all locations have the necessary infrastructure to support cold-chain delivery. The cost and logistical challenges associated with cold storage can be substantial, particularly in remote or resource-limited settings such as in developing nations, space missions or military operations.^7,34^ Therefore, alternative strategies are necessary to enable the use of synthetic cells in these environments.

Since synthetic cells stored at room temperature (with encapsulated TXTL and DNA) would activate before arriving at the point of use (Fig. S4, ESI†), we attempted to store them in a dried state. To keep costs as low as possible, we followed the approach employed by Guzman-Chavez *et al.* and employed a low-cost silica-drying device as an alternative to high-cost commercial freeze-dryers^35^ ($50 *vs.* thousands of dollars).

We prepared synthetic cells and dried them using a low-cost silica-drying device overnight. The synthetic cells were prepared and dried with an external sucrose solution since it provided the best results at 4 °C, at which drying took place to prevent undesired TXTL activation. The dried samples were subsequently stored at room temperature, and their activity was evaluated upon rehydration at different time points (Fig. 2). The synthetic cells remained active after one week when stored in a dried state at room temperature. After rehydration, the GFP fluorescence was lower than that from fresh samples, which could be attributed to lower expression due to the exchange of contents between the lumen and the surroundings upon rehydration.^29,33,36^ Sugars offer dual protection; during drying, the sugar molecules replace the water and form stabilising hydrogen bonds with the lipid headgroups while forming a highly viscous matrix surrounding the synthetic cell that prevents vesicle destabilisation.

**Fig. 2:**
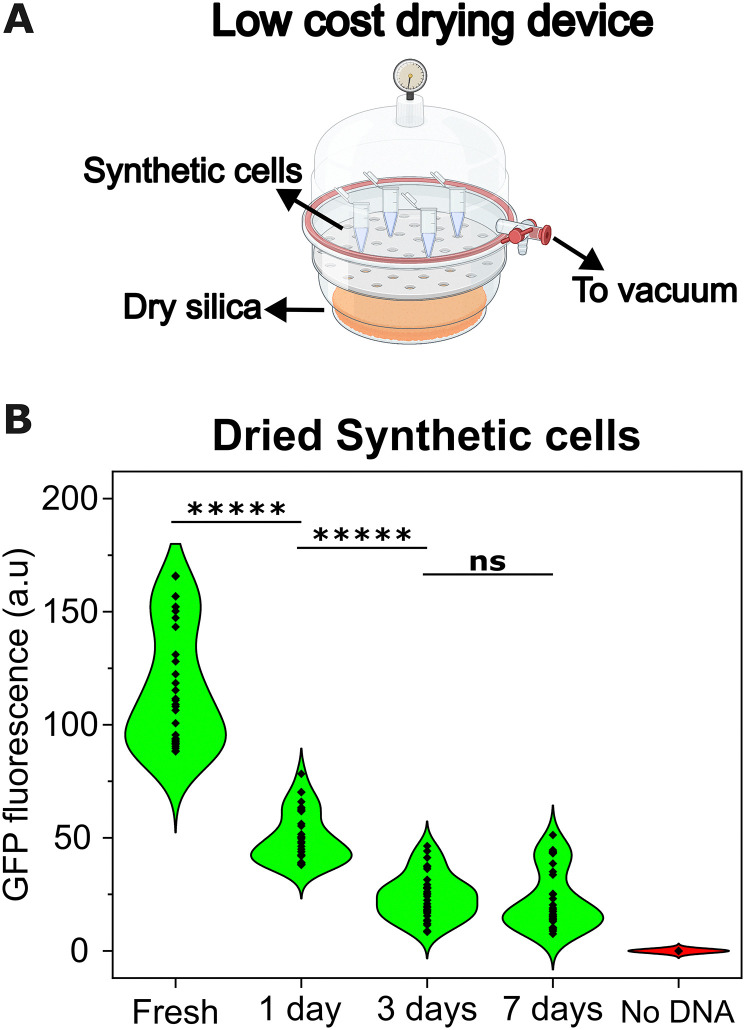
Synthetic cells can be preserved at room temperature in a dried state. (A) Schematic of the low-cost drying device employed. Synthetic cells were prepared and dried overnight at 4 °C in a desiccator filled with dry silica beads and connected to a 0.1 mbar vacuum line. (B) Violin plot showing the distribution in fluorescence intensities obtained from microscopy images of over 50 synthetic cells kept dry for up to a week, after rehydration and incubation. Statistical analysis of GFP fluorescence was performed using an unpaired two-tailed *t*-test assuming unequal variance. *P* value: ***** <0.0005, *** <0.0005, ** <0.005, * <0.05, ns = not significant.

To confirm that no protein expression occurred during the 7-day storage period prior to rehydration, GFP fluorescence was measured immediately after rehydration. The signal was minimal compared to the significantly higher fluorescence observed following incubation at 37 °C post-storage, indicating that protein expression did not activate during the period where synthetic cells were being stored (Fig. S5, ESI†).

One aspect that emerges from this research is whether the encapsulation of TXTL machinery inside the synthetic cell presents an advantage over bulk, unencapsulated expression. On top of all the possibilities that synthetic cells offer for medical applications for example—such as serving as delivery vehicles^6^ with functionalised membranes^37^ and incorporating stimuli-responsive features for enhanced targeting^3,38^ – one key aspect to consider is that the lipid membrane acts as a physical barrier that prevents exogenous agents from affecting cell-free protein expression. To demonstrate this, we added enzymes that disrupt protein synthesis to TXTL in bulk (Fig. 3A) or to the outside of synthetic cells (Fig. 3B).

**Fig. 3:**
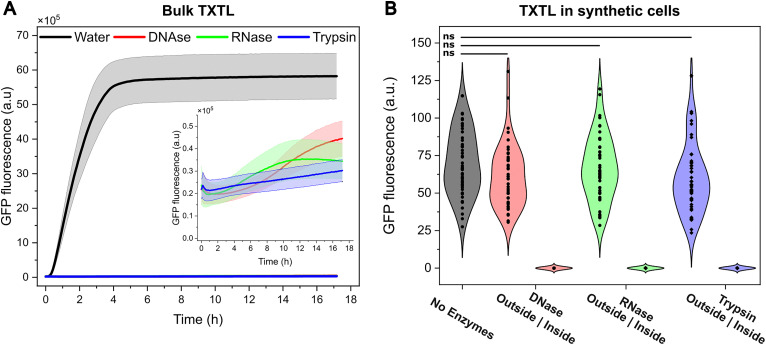
The lipid membrane of the synthetic cell protects the encapsulated TXTL machinery from exogenous disruptive agents. (A) Protein expression in bulk is sharply affected by the presence of the disruptive agents DNAse, RNase, and Trypsin. The shaded area represents 1 SD (*n* = 3 independent experiments). (B) Violin plots showing the distribution in GFP fluorescence recorded from microscopy images of over 50 synthetic cells incubated for 2 h with enzymes present outside or inside them (left | right distributions). When the TXTL is shielded inside the synthetic cells, enzymes added to their exterior do not affect the level of GFP expression. Statistical analysis of GFP fluorescence was performed using an unpaired two-tailed t-test assuming unequal variance. ns = not significant.

Enzymes degrading the DNA, RNA or proteins (DNase, RNAse, Trypsin) cause a 100-fold reduction in protein expression when the TXTL machinery is in bulk (panel A). However, when TXTL is encapsulated inside synthetic cells and the enzymes are in the exterior, there is no significant reduction in expression (panel B, unpaired *t*-test, *n* > 50) compared to an enzyme-free scenario. In contrast, the co-encapsulation of such enzymes with TXTL inactivates it, demonstrating that the lipid membrane acts as a protective barrier for the encapsulated TXTL.

Future research could focus on enhancing the formulation stability beyond 7 days by incorporating lipid mixtures that are more resistant to phase transitions and alternative cryoprotectants, optimising the preservation process, and integrating more advanced genetic components that enable sophisticated functionalities.^5,6,39,40^ Beyond the potential to activate synthetic cells at the point of use, we have also demonstrated that the lipid membrane provides enhanced protection, shielding the TXTL system from external molecules that would otherwise inhibit or disrupt expression. Taken together, this opens new possibilities for using TXTL systems outside of controlled laboratory environments, facilitating applications in biosensing, biomanufacturing, and therapeutics. In these contexts, TXTL systems may encounter various substances in solution that could hinder their functionality. In the absence of laboratory resources, such as purification equipment to counteract the above, the protective capabilities of lipid membranes are critical for preserving the system's activity and ensuring reliable performance in practical applications. Additionally, this approach offers a potential pathway for scalable biomanufacturing by simplifying the production and distribution of synthetic cells at scale, a complementary approach to point-of-use synthetic cell synthesis using portable lab-on-chip systems, for example.^41^

In conclusion, our results constitute a proof of concept that synthetic cells could be employed at the point of use with distribution *via* cold-chain or room-temperature transportation, making them more accessible for clinical and industrial use.

This work was supported by a UKRI Future Leaders Fellowship, grant reference number MR/S031537/1 (awarded to Y. E.) as well as BBSRC grant BB/W00125X/1.

## Data availability

The data supporting the findings of this study are available within the article and its ESI.†

## Conflicts of interest

There are no conflicts to declare.

## Supplementary Material

CC-061-D5CC00826C-s001
