# Supplementary material for: Synthetic cell preservation strategies enable their storage and activation at the point of use
Source: Chem Commun (Camb). 2025 Apr 29;61(46):8359–62. doi: 10.1039/d5cc00826c (PMC12070246; doi:10.1039/d5cc00826c)
Supplement: CC-061-D5CC00826C-s001 [file CC-061-D5CC00826C-s001.pdf]

Supplementary Information (SI) for ChemComm.
This journal is © The Royal Society of Chemistry 2025

Supplementary information

Synthetic cell preservation strategies enable their storage and activation at the point of use

Ignacio Gispert [a,b] and Yuval Elani [a,b]*

[a.] Department of Chemical Engineering, Imperial College London, South Kensington, London SW7 2AZ, U.K.

[b.] fabriCELL Imperial College London, South Kensington, London SW7 2AZ, U.K.

* y.elani@imperial.ac.uk

<u>**Supplementary Methods**</u>

Synthetic cell formation

Our protocol was adapted from Monck *et al*[1]. Synthetic cells were formed using the emulsion phase transfer method. First, 10 mg of EggPC lipid (Avanti Polar Lipids) was dissolved in chloroform, which was then evaporated off using a gentle stream of nitrogen gas to form a lipid film. This was left overnight in a desiccator to ensure complete chloroform removal. Then, the lipid was dissolved in 1 mL of mineral oil with the aid of 30 min of sonication at 40 °C to allow complete dissolution. An Eppendorf containing 300 µL of 0.5 M glucose was prepared and 150 µl of lipid-in-oil were layered on top and left to sit while the inner components' emulsion was prepared. The PURExpress reaction consisting of 10 µL of solution A, 7.5 µL of solution B, 0.5 µl of murine RNAse inhibitor (NEB) and 250 ng of DNA was supplemented with 4 µL of 2 M maltotriose and mixed with 250 µl of the lipid-in-oil in an empty Eppendorf tube and a stable emulsion was formed by pipetting. The emulsion containing the TXTL machinery was gently layered onto the glucose-layered oil Eppendorf previously produced. The synthetic cells were formed by centrifugation at 8.0 r.c.f. for 30 min at 4 °C. The supernatant was later removed, and the pellet was resuspended in 300 µL of 0.8 M fresh sugar solution before subsequent treatment.

Cell-free protein synthesis

PURExpress In Vitro Protein Synthesis Kit (New England Biolabs (NEB)) was chosen for cell-free TXTL protein synthesis. It was assembled following the NEB protocol using 0.5 µl of murine RNAse inhibitor (NEB) and 250 ng of dasherGFP plasmid (synthesised by DNA 2.0 and

[1] C. Monck, Y. Elani and F. Ceroni, *Nat. Chem. Biol.*, 2024, **20**, 1380–1386.

containing an *E. coli* pJexpress 441 vector with T7 promoter and terminator sequences). Activation of protein expression took place at 37 ^oC and synthetic cells were imaged after 2 h of incubation in a thermocycler. For dynamic measurements of GFP production over time, samples were incubated at 37 ^oC in a CLARIOstar Plus plate reader (BMG Labtech) with fluorescence recordings every 5 (λex/em 470-15/515-20). For experiments to demonstrate the protective barrier effect conferred by the synthetic cell membrane, 1 U of DNAse or 10 U RNAse was employed (enough to degrade 4 times the amount of DNA initially added). Trypsin was also added in excess, 1000 U were employed.

Synthetic cell storage

Synthetic cells were kept on ice at all times during handling. For cold storage, they were stored in the fridge, freezer or ultra-low temperature freezer (4, -20, -80 ^oC) overnight before being retrieved. They were then incubated at 37 ^oC for 2 h, which activated the expression of GFP.

Low-cost drying was possible thanks to a low-cost silica-drying device consisting of a silica-filled desiccator connected to a gentle vacuum pump (0.1 mbar). Synthetic cells were placed in eppendorfs which were left open overnight in the desiccator for the samples to dry. Drying took place in a cold room (4 ^oC) to prevent TXTL activation during drying.

Synthetic cell visualisation and analysis

Synthetic cells were visualized on a Nikon eclipse Ti2-U inverted microscope with a CoolLED pE-300white and a Nikon DS-Qi2 camera with a FITC-3540C filter cube to image GFP fluorescence. The intensity of fluorescence images was analysed using ImageJ and Excel and normalised by removing the local background signal for each sample. Statistical analysis of GFP fluorescence was performed using an unpaired two-tailed t-test assuming unequal variance ($\alpha = 0.05$). P value: **** <0.0001, *** <0.0005, ** <0.005, * <0.05. The standard deviation was calculated assuming the recorded values to be a sample of the whole population.

Supplementary Figures

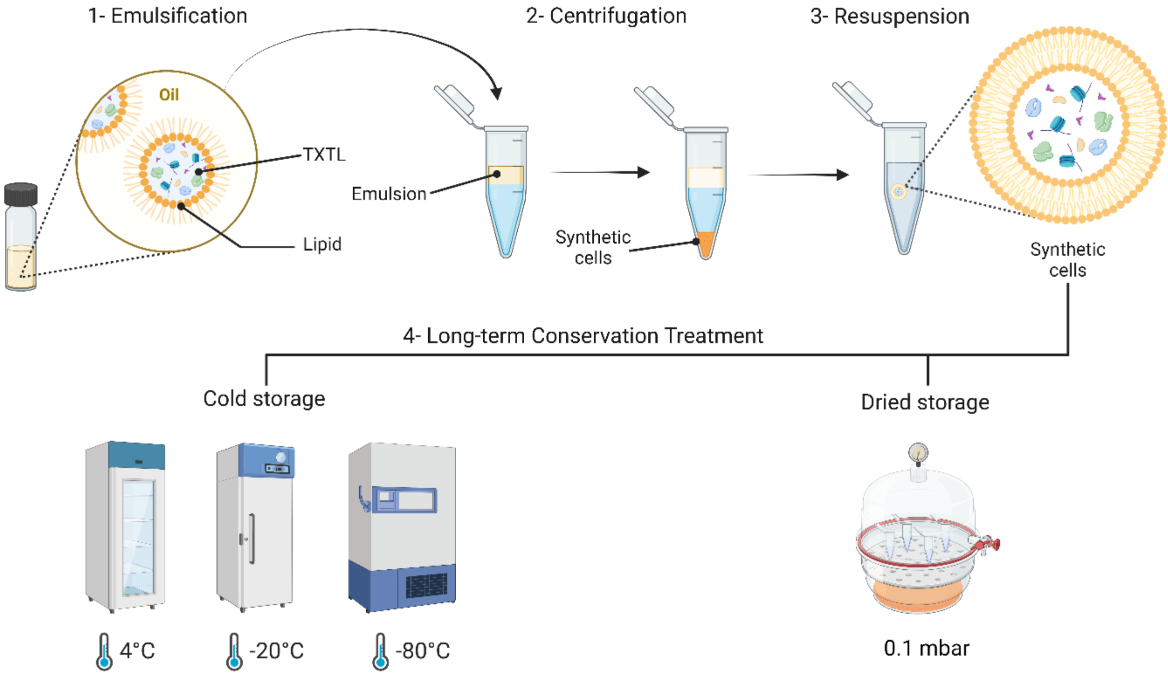

Supplementary Figure 1.- Schematic of the preparation and long-term conservation of synthetic cells encapsulating TXTL machinery*. First, an emulsion is formed using the TXTL machinery and EggPC lipids dissolved in mineral oil. The emulsion is then layered over an aqueous solution, and the synthetic cells are collected by centrifugation. After resuspension in an appropriate cryoprotective solution, the synthetic cells are stored under cold or dry conditions.*

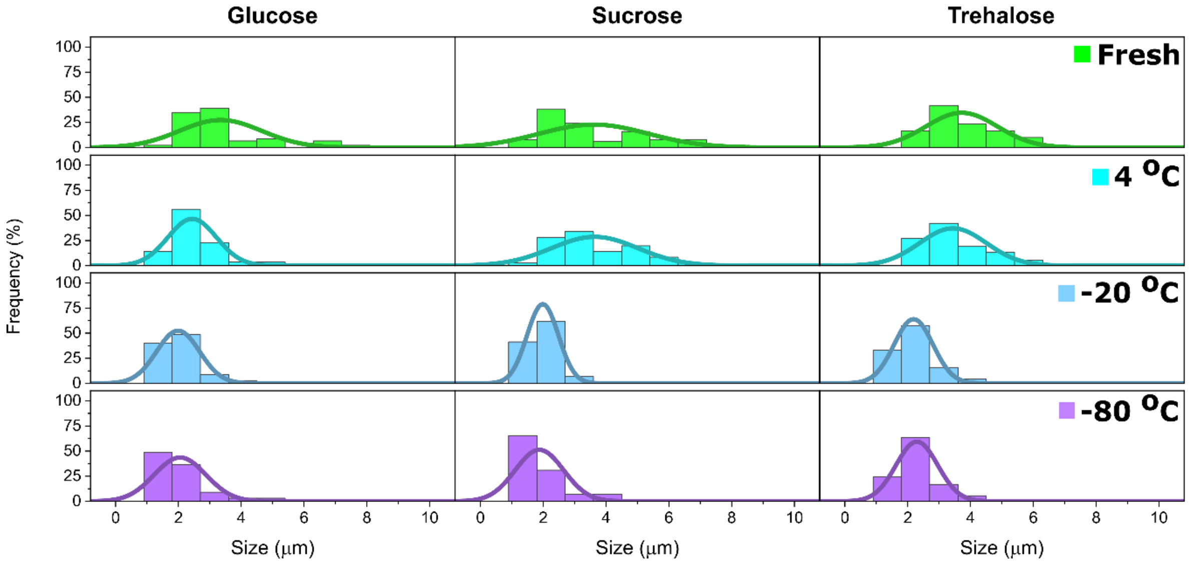

Supplementary Figure 2.- Analysis of the effects of cold storage on the size of the Synthetic Cells. *The radius of synthetic cells stored in glucose were smaller than just after production, whereas sucrose and trehalose only saw reductions in radius when the samples were frozen at -20 and -80 °C. This data, in combination with the activation recordings of Figure 1, indicates that sucrose provided the best protection out of the three sugars.*

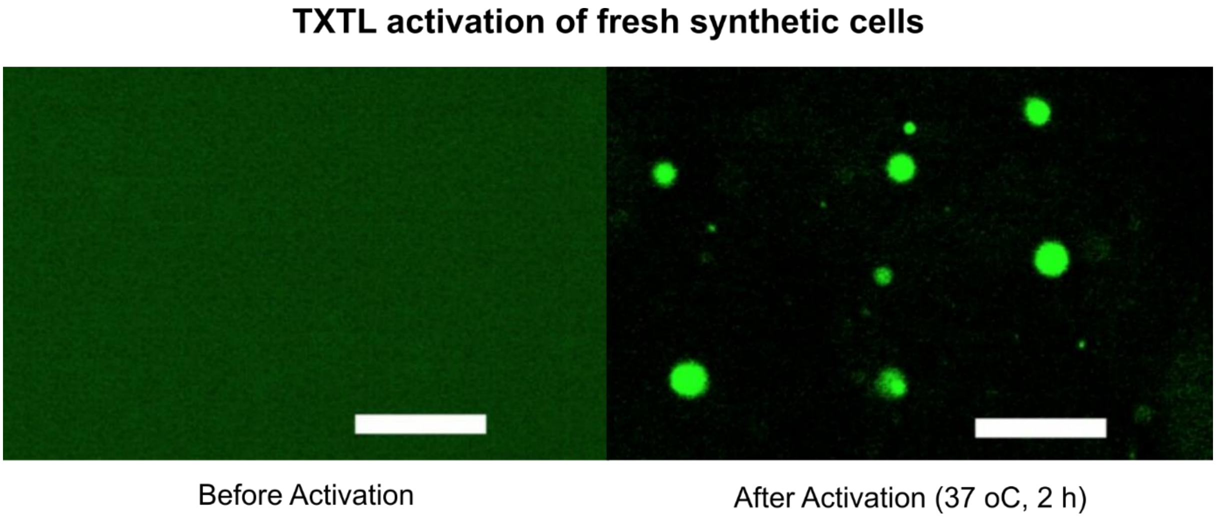

Supplementary Figure 3. Synthetic cells do not activate before the preservation process*. Fluorescent microscopy images of synthetic cells before (left) and after (right) incubation at 37 °C for 2 h. GFP can only be observed after incubation. Before this point (i.e., directly after they are generated, during the preparation method, and immediately before they are cooled/frozen), there is no sign of TXTL activation (left). This confirms that minimal GFP expression was present at the point of preservation. Therefore, the observed GFP expression following incubation after retrieval from cold or frozen storage is not a false positive and cannot be attributed to residual expression from the initial vesicle preparation at room temperature prior to preservation. Size bar indicates 20 μm.*

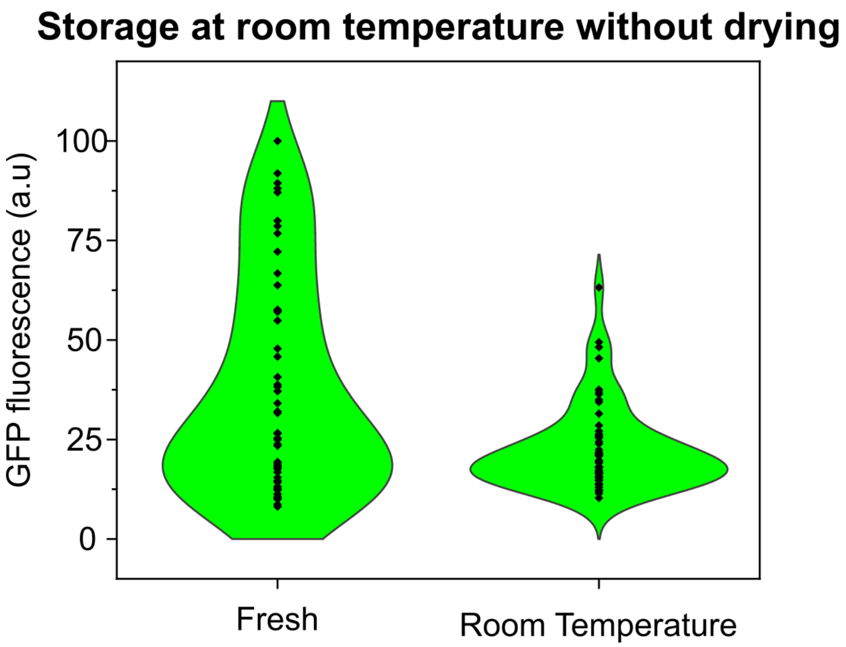

Supplementary Figure 4.- Untreated synthetic cells stored at room temperature overnight activate spontaneously. *Fluorescence microscopy image analysis revealed that synthetic cells stored at room temperature overnight (right) expressed GFP at 60% of the maximum signal observed in freshly prepared samples incubated for 2 h at 37°C immediately after manufacture (left). This highlights one reason for drying synthetic cells: to enable room-temperature storage and distribution, which prevents them being activated before their intended time of use.*

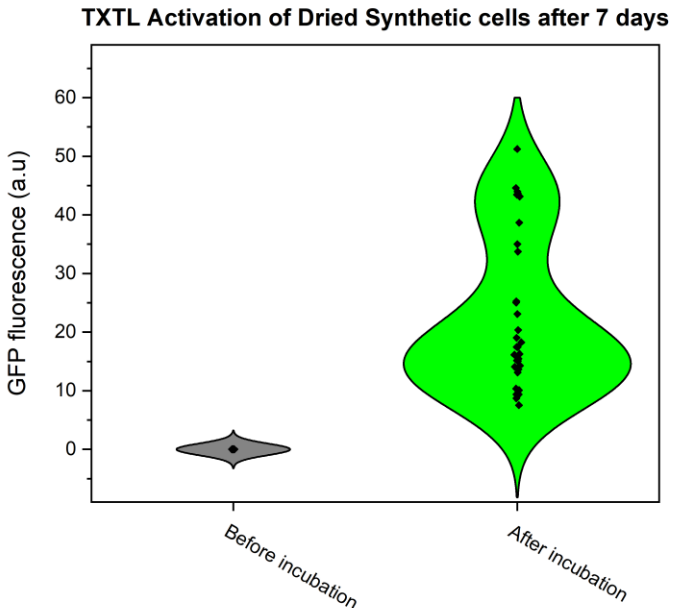

Supplementary Figure 5.- Dried synthetic cells stored at room temperature do not express protein while in a dried state over 7 days. *Fluorescence microscopy image analysis of rehydrated synthetic cells revealed that drying suppressed the expression of GFP (left) when the samples were stored at room temperature. Immediately after rehydration, but before incubation, only minimal fluorescence was observed. Upon incubation for 2 h at 37°C the encapsulated TXTL in the dried synthetic cells activated and produced GFP (left). This shows that protein expression did not activate during the period where synthetic cells were being stored, but only after, when they were hydrated and incubated at elevated temperature.*
